# Amelioration of Cancer Stem Cells in Macrophage Colony Stimulating Factor-Expressing U87MG-Human Glioblastoma upon 5-Fluorouracil Therapy

**DOI:** 10.1371/journal.pone.0083877

**Published:** 2013-12-31

**Authors:** S. Chockalingam, Siddhartha Sankar Ghosh

**Affiliations:** 1 Department of Biotechnology, Indian Institute of Technology Guwahati, Guwahati, Assam, India; 2 Centre for Nanotechnology, Indian Institute of Technology Guwahati, Guwahati, Assam, India; University of Colorado Denver, United States of America

## Abstract

Macrophage colony stimulating factor (MCSF) regulates growth, proliferation and differentiation of haematopoietic cell lineages. Many cancers are known to secrete high level of MCSF, which recruit macrophages into the tumour micro-environment, supporting tumour growth. Herein, we report the cloning of MCSF and subsequent generation of U87MG expressing MCSF stable cell line (U87-MCSF). Cytotoxicity of anti-cancer drug 5-fluorouracil (5-FU) was evaluated on both U87MG and U87-MCSF cells. Interestingly, the proliferation of U87-MCSF cells was less (p<0.001) than that of U87MG cells alone, after treatment with 5-FU. Significant decrease in expression levels of cyclin E and A2 quantified by real time PCR analysis corroborated the reduced proliferation of 5-FU treated U87-MCSF cells. However, JC-1 staining did not reveal any apoptosis upon 5-FU treatment. Notch-1 upregulation induced a possible epithelial-mesenchymal transition in U87-MCSF cells, which accounted for an increase in the proportion of CD24^high^/CD44^less^ cancer stem cells in U87-MCSF cells after 5-FU treatment. The elevated resistance of U87-MCSF cells towards 5-FU was due to the increase in the expressions (10.2 and 6 fold) of ABCB1 and mdm2, respectively. Furthermore, increase in expressions of ABCG1, mdm2 and CD24 was also observed in U87MG cells after prolonged incubation with 5-FU. Our studies provided mechanistic insights into drug resistance of U87MG cells and also described the pivotal role played by MCSF in augmenting the resistance of U87MG cells to 5-FU.

## Introduction

Macrophage colony stimulating factor (MCSF), also referred to as colony stimulating factor-1(CSF-1), is a growth factor responsible for survival, proliferation and differentiation of cells of hematopoietic lineages [1]. Outside the hematopoietic system, MCSF has an important role in the development and regulation of placenta, mammary gland, brain and bone physiology [2]–[4]. MCSF is encoded by a unique gene, however, through alternative mRNA splicing and differential post-translational modification, three different forms of MCSF, such as, a secreted glycoprotein, a secreted proteoglycan and a short membrane bound isoform are found [1]. MCSF acts through a type III tyrosine kinase receptor, colony stimulating factor 1 receptor (CSF1R), which is the product of c-fms proto-oncogene.

MCSF is known to infiltrate sites of injury and inflammation with mononuclear phagocytes. Homozygous null mutation of CSF-1 in mice shows a depleted macrophage population in breast cancer, resulting in reduced malignancy and metastasis [5]. The presence of monocytes and macrophages promotes angiogenesis and metastasis in tumor by increasing the level of secretion of vascular endothelial growth factor (VEGF). MCSF acts as a transcriptional regulator for production of VEGF [6]. Nevertheless, MCSF has a potential role in eliciting anti-tumor response. Monocytes and macrophages have been reported to kill cancerous cells by paraptosis, with overexpression of membrane form of MCSF [7], [8]. Addition of purified MCSF to the human ovarian cancer cells has been documented to induce concentration dependent growth inhibition in vitro [9]. Hence, such reports demonstrating anti-tumor activities of MCSF run hand-in-hand with alternative reports showing the pro-tumoral properties of MCSF.

In this study, we have elucidated the role played by MCSF in increasing the drug resistive properties of human glioblastoma cell line, U87MG. We also found the mechanism of 5-FU resistance in U87MG cells. Our results illustrated that Notch-1 expression was enhanced in untreated U87-MCSF cells, which induced epithelial-mesenchymal transition. An increase in CD24^high^/CD44^low^ cancer stem cells and upregulation of key ABC transporter genes (ABCG1 and ABCB1) imparted resistance to 5-FU in U87-MCSF cells. Our data provides evidence for the drug resistant phenotype emerging through the formation of cancer stem cells in MCSF expressing glioblastoma.

## Materials and Methods

### Cell lines

ACHN, human renal carcinoma and U87MG, human glioblastoma cell lines procured from National Centre for Cell Science, Pune were maintained in Dulbecco's Modified Eagle's medium (DMEM) supplemented with 10% Fetal Bovine Serum, Penicillin (50 U/ml)-Streptomycin (50 mg/ml) at 5% CO_2_ in a humidified incubator at 37°C.

### RNA isolation and RT-PCR

RNA from cultured mammalian cells was isolated by using GenElute mammalian total RNA isolation kit (sigma) as per manufacturer's instructions. Total RNA (1 µg) was reverse transcribed using cDNA synthesis kit (Fermentas). Amplification of gene expression was performed with respective gene specific primers (Table S1 in File S1).

### Western blotting

Cells grown to 70–80% confluency were lysed by RIPA buffer containing 1 mM PMSF. Total protein content in the cell lysates was quantified by Lowry's method of protein estimation using BSA as standard. SDS-PAGE was done loading equal amount of protein in each well. The samples were blotted onto PVDF membrane and detected using antibodies for β-actin (BD Transduction Laboratories) and MCSF (Sigma). Blots were developed using chemiluminescent peroxidase substrate-1 kit (Sigma) and imaged using gel documentation system (Molecular imager ChemiDoc XRS+ image system, Bio-Rad).

### Cell viability assay

Cell viability was measured by using In Vitro Toxicology assay kit, XTT based (Sigma). Cells seeded in 96 well microplate at a density of 1.5×10^3^ cells per well were allowed to grow overnight and then treated with various concentrations of 5-FU for different time intervals. XTT (2, 3-bis [2-Methoxy-4-nitro-5-sulfophenyl]-2H-tetrazolium-5-carboxyanilide inner salt) assay was performed at the end of treatment period using manufacturer's protocol. The soluble orange formazan product was measured using multiplate reader (Tecan, Infinite M200) at 450 nm and the background measurement at 690 nm. Cell viability (%) was calculated relative to untreated 100% viable cells.

### MCSF Localisation study

Briefly, cells were washed with PBS and fixed with 3.7% paraformaldehyde solution (with or without 0.1% Triton X-100) at 37°C for 30 min. After washing with PBS for three times, cells were blocked with 1% BSA blocking solution for 30 min. Then, cells were incubated with anti-MCSF (Sigma) primary antibody and subsequently with FITC tagged secondary antibody (BD Transduction Laboratories). After staining with FITC tagged secondary antibody, cells were incubated with media containing 300 nM DAPI for 3 min, finally washed and visualised under fluorescence microscope (Nikon ECLIPSE T*i*-U, Japan) with an excitation filter of 480/15 nm (for FITC) and 360/20 nm (for DAPI).

### Trypan blue dye exclusion assay

Cells in six well plate were treated with 5-FU for 120 h. After treatment, cells were harvested, mixed with equal volume of 0.4% trypan blue (Invitrogen) and loaded over a counting chamber. The healthy and viable cells with intact membrane excluded the dye, whereas compromised cells stained with the dye and were counted as dead. The percentage (%) of cell viability was calculated by using Countess-automated cell counter (Invitrogen).

### CFSE cell proliferation assay

Cells (1×10^6^ cells/ml) in PBS containing 0.1% BSA were incubated with 10 µl of 0.5 mM CFDA-SE at 37°C for 10 min and then washed with 5 volumes of ice-cold media to quench any free dye. Cells were collected by centrifugation and washed twice with fresh media. Stained cells were analysed immediately by flow cytometer (zero time point) and the rest of the cells were seeded for subsequent time periods. The data acquired were analysed in Cell Quest Pro software. The doubling time was calculated according to the formula T_d_  =  T/log_2_ (F_0_/F_T_), where F_0_ is the geometric mean fluorescence intensity at 0 h and F_T_ is the geometric mean fluorescence intensity at T h [10]. In our experiments T was 72 h.

### Cell cycle analysis

Cells were seeded in six well plates at density of 5×10^4^ cells per well. After overnight attachment, cells were treated with different concentrations of 5-FU. At the end of the treatment period cells were trypsinised, washed and fixed with 70% alcohol solution for 15 min in ice. The fixed cells were stained with propidium iodide (PI) staining solution (50 µg/ml PI, 0.1 mg/ml RNase A and 0.05% triton X-100) at 37°C for 30 min in dark. At least 10000 events per sample were acquired by flow cytometer and the percentage of cells distributed in different phases of cell cycle was calculated using ModFit LT software.

### CD24/CD44 analysis by flow cytometry

Cells after treatment were dissociated by trypsinization, washed twice with PBS and incubated with 10% human serum for 20 min on ice to block Fc receptors. FITC Mouse Anti-Human CD24 and PE Mouse Anti-Human CD44 (both from BD Pharmingen) antibodies were added and incubated for 20 min on ice in the dark. The cells were washed twice with PBS, finally resuspended in PBS and analysed by a flow cytometer (FacsCalibur, BD Biosciences, NJ).

### Gene expression analysis by real time PCR

Quantitative real time PCR was performed using SYBR Green as reporter dye (Power SYBR Green PCR master mix, Applied Biosystem) and 7500 Real time PCR system (Applied Biosystem). Raw data was analysed and efficiency of each reaction was calculated by LinRegPCR software. β-actin was used as the endogenous control and the fold change in expression of genes was calculated by ΔΔCt method.

### Methylene blue staining

Cells treated with various concentrations of 5-FU for different time intervals, were washed with ice-cold PBS, fixed with 50% ice-cold ethanol. 0.2% (W/V) methylene blue staining solution was added to the plate and staining was carried out for 30 seconds. The solution was aspirated and the cells were washed thrice with ice-cold water. Samples were air dried and visualised under a light microscope.

### Actin cytoskeleton staining

Cells seeded in six well plates were treated with 5-FU. At the end of treatment period, cells were washed with PBS and fixed with 3.7% paraformaldehyde solution containing 0.1% Triton X-100 at 37°C for 30 min. After washing with PBS for three times, cells were blocked with 1% BSA blocking solution for 30 min. Then, cells were incubated with anti β-actin primary antibody (BD Transduction Laboratories) and subsequently with FITC tagged secondary antibody (BD Transduction Laboratories). After staining with FITC tagged secondary antibody, cells were washed thrice and incubated with media containing 300 nM DAPI for 3 min. Cells were washed thoroughly, finally resuspended in PBS and visualised under fluorescence microscope (Nikon ECLIPSE T*i*-U, Japan) with an excitation filter of 480/15 nm (for FITC) and 360/20 nm (for DAPI).

### Statistical analysis

The values for all experiments were expressed as mean ± s.e.m of three or more individual experiments. The data were analysed by Student's t test or by ANOVA whichever applicable, using GraphPad Prism 5.01. Statistically significant values are denoted by * (p<0.05), ** (p<0.01) and *** (p<0.001).

## Results

### Generation of U87-MCSF cells and sensitivity towards 5-FU

**Figure 1:**
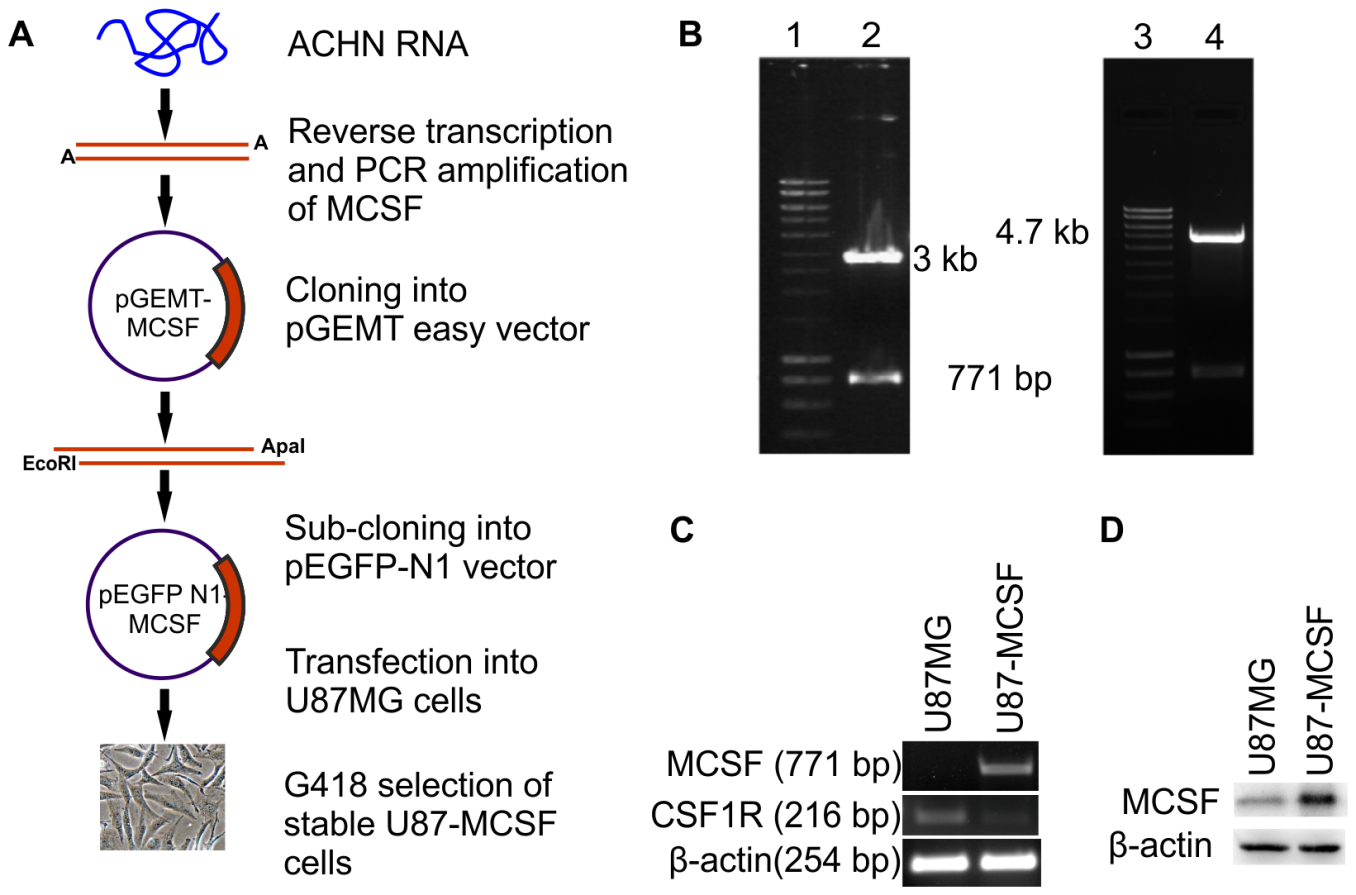
Cloning and generation of U87-MCSF cell line. A. Schematic for generation of U87-MCSF cell line. B. Confirmation of clones by restriction digestion Lanes 1 and 3: 1 kb DNA ladder, lane 2: pGEMT-MCSF digested with EcoR I, lane 4: pEGFP-N1-MCSF digested with EcoR I and Apa I. C. RT-PCR analysis for expression of MCSF and CSF1R in U87MG and U87-MCSF cells. D. Western blot analysis of MCSF protein. A 28 kD band confirmed the overexpression of membrane bound MCSF in U87-MCSF cells.

Figure 1A depicted the strategy for generation of U87-MCSF stable cell line. The 0.771 kb PCR amplified gene corresponding to membrane bound isoform of MCSF was cloned sequentially into pGEMT-easy and pEGFP-N1 vectors. The clones were checked by restriction analysis in Figure 1B (lane 2 and 4). A stable cell line (U87-MCSF), overexpressing MCSF was established by transfecting U87MG cells with pEGFP-N1-MCSF. A mixed population of clones of U87-MCSF was selected with G418 (400 µg/ml) to exclude the potential role of any other compensatory process arising out of transfection in a single clonal population. The expression of transgene MCSF was confirmed by semi-quantitative RT-PCR (Figure 1C). The overexpression of MCSF protein was confirmed by western blotting using anti-MCSF antibody by which, a 1.8 fold increase in MCSF expression was noted in U87-MCSF cells (Figure 1D). A decrease in the expression of MCSF receptor, CSF1R was noted in U87-MCSF cells (0.16 fold expression in U87-MCSF cells as compared to U87MG cells) (Figure 1C).

**Figure 2:**
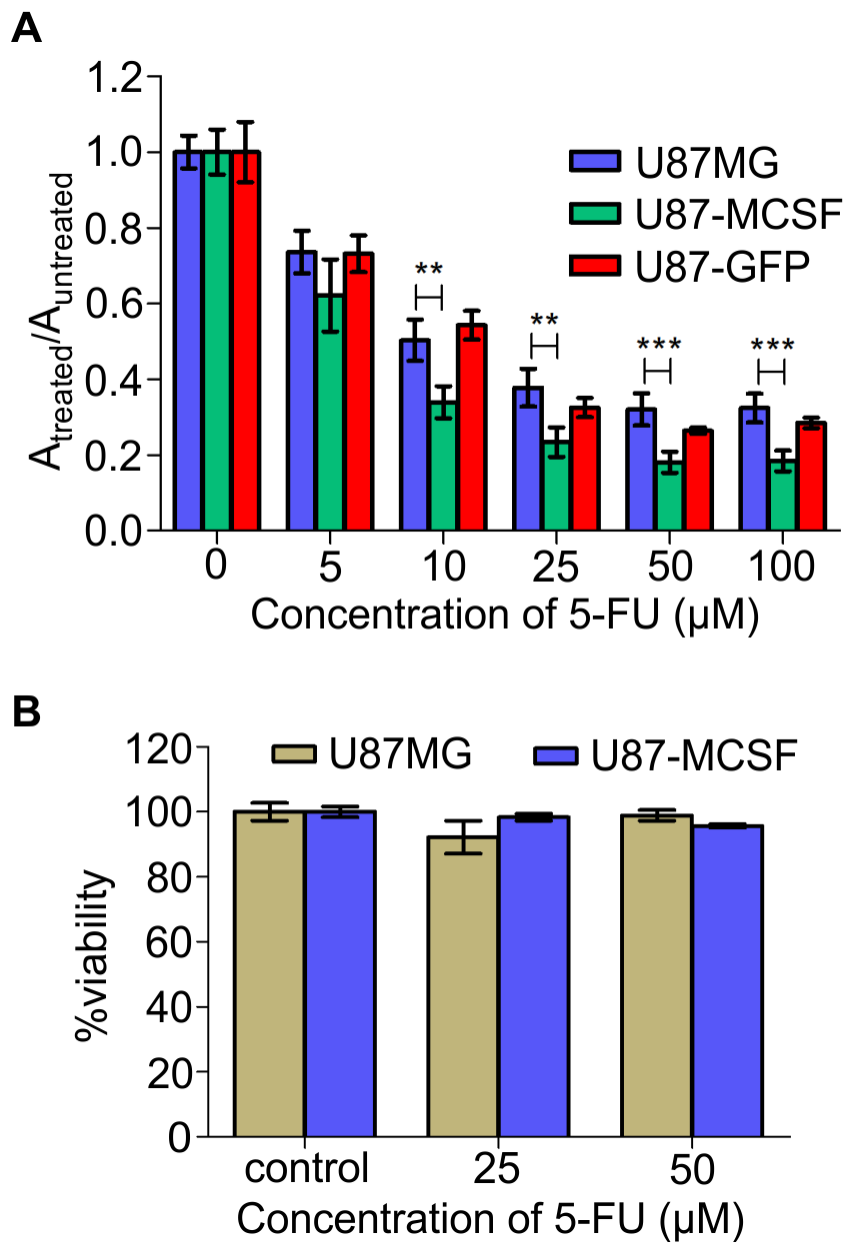
5-FU treatment of U87MG, U87-MCSF and U87-GFP cells. A. Effect of 5-FU on growth and viability of U87MG, U87-MCSF and U87-GFP cells calculated by XTT assay after 72 h of 5-FU treatment. B. Trypan blue dye exclusion assay showed healthy populations after 120h of 5-FU treatment. Statistical significance is denoted by * (p<0.05), ** (p<0.01) and *** (p<0.001).

Sensitivity of U87-MCSF cells was checked by XTT assay after treatment with various concentrations of 5-FU ranging from 5-100 µM, for 72 h. The proliferation of U87-MCSF cells was significantly reduced with 5-FU of above 10 µM as evident from the reduced absorbance values (Figure 2A). The similar growth reduction was not observed in U87-GFP cell line with 5-FU treatment, where the proliferation was almost similar to that of treated U87MG cells. Additionally, no indication of apoptosis was seen in treated U87MG or treated U87-MCSF cells after JC-1 staining (Figure S1 in File S1). The results were substantiated with quantitative trypan blue exclusion assay which showed healthy cell populations with intact cell membrane after 120 h of treatment (Figure 2B). We investigated the expression of some of the pro-apoptotic and anti-apoptotic genes like caspase-3, Bax and Bcl-xL. As shown in Figure S2 in File S1, the expression of pro-apoptotic gene, Bax was increased in treated samples of both U87MG (1.90 fold) and U87-MCSF cells (1.29 fold). The expression of anti-apoptotic gene, Bcl-xL was also increased in treated U87MG and treated U87-MCSF cells. However, the increase in Bcl-xL expression was 2.11 fold in treated U87-MCSF cells as compared to the 1.25 fold increase in treated U87MG cells. Caspase-3 expression remained unchanged in treated samples of U87MG and U87-MCSF cells.

### Effect of 5-FU on cell cycle and cyclins

**Figure 3:**
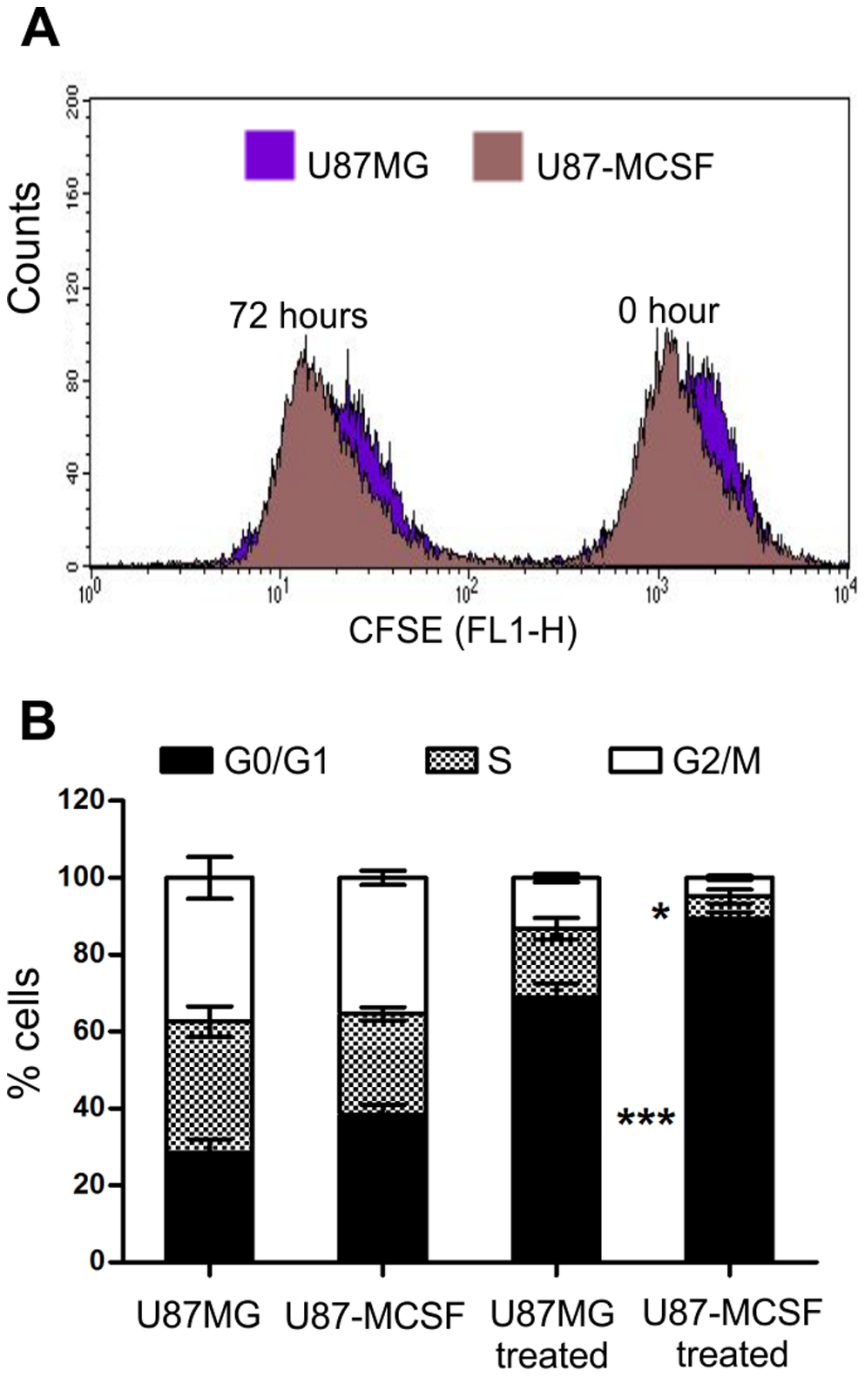
Proliferation and cell cycle analysis of U87MG and U87-MCSF cells. A. CFSE cell proliferation assay showed that the rate of proliferation of untreated U87MG and U87-MCSF cells remained unaltered. B. Cell cycle analysis of U87MG and U87-MCSF cells after treatment with 5-FU for 24 h. Majority of cells were accumulated in G0/G1 phase in treated population of U87-MCSF cells. Statistical significance is denoted by * (p<0.05), ** (p<0.01) and *** (p<0.001).

The cell cycle upon 5-FU treatment was studied by propidium iodide staining using flow cytometer. Initially, both U87MG and U87-MCSF cells were synchronised in G0/G1 phase by serum starvation for 48 h and then the cell cycle pattern was observed in complete serum containing media at regular time intervals. The rate of progression of cell cycle between two cell lines was same (Figure S3 in File S1). This was also confirmed by CFSE cell proliferation assay (Figure 3A) by which the doubling time of both the cell lines was found to be 12 h.

Next, pre-synchronised cells were treated with 25 µM 5-FU in serum containing media for 24 h. The cell cycle distribution pattern analysed by flow cytometer showed significantly higher percentage (90%) of treated U87-MCSF cells in G0/G1 phase than that of treated U87MG (69%) cells (Figure 3B). This was accompanied by a corresponding decrease in proportion of cells in S and G2/M phases. Comparatively, 75% of U87-GFP cells were accumulated in G0/G1 phase upon 5-FU treatment (data not shown).

**Figure 4:**
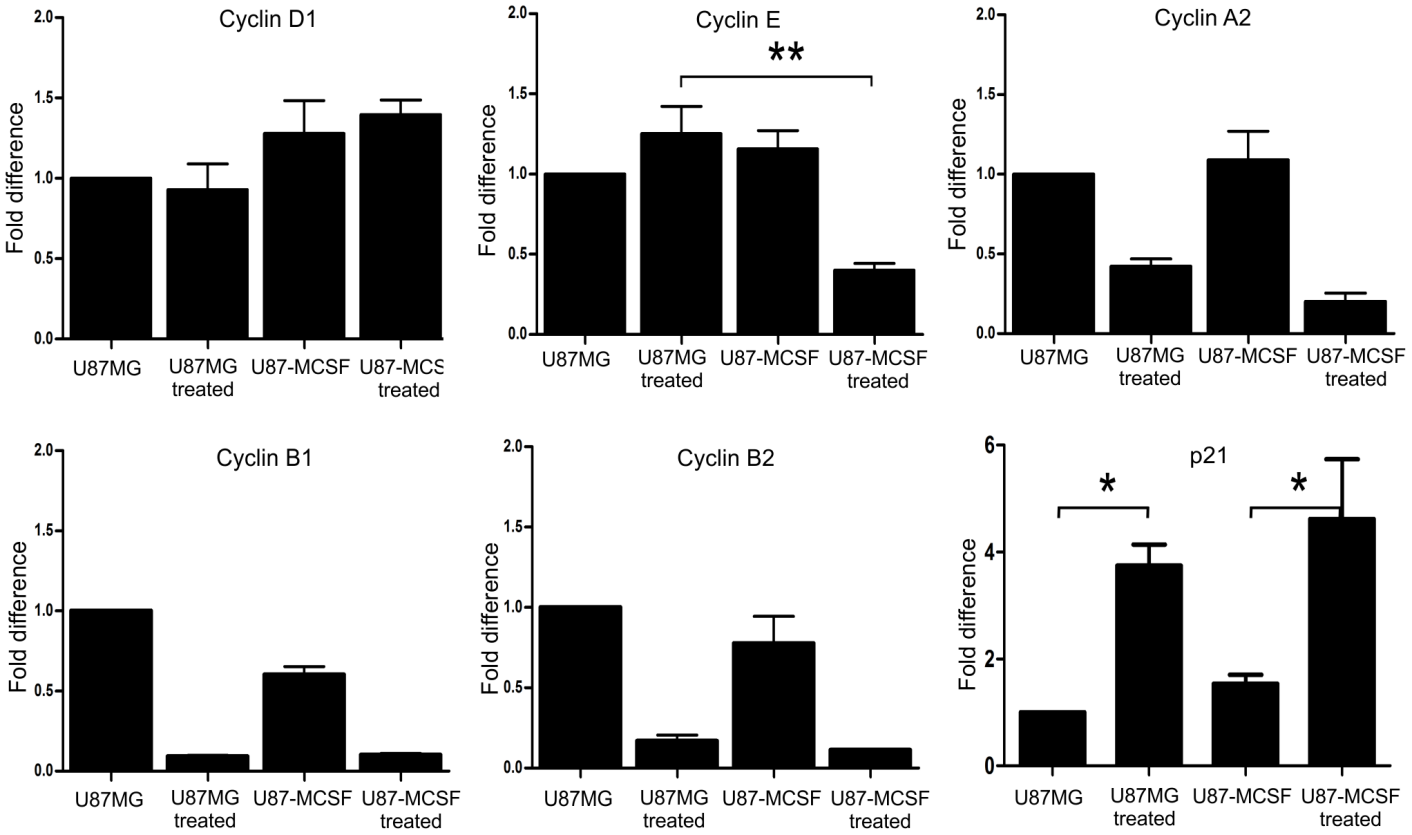
Real time PCR analysis to show the expression levels of various cyclins and p21. A significant decrease in the expression of cyclin E and a slight decrease in the expression of cyclin A2 was observed in treated U87-MCSF cells, which correlated with the elevated G0/G1 accumulation of cells seen in cell cycle analysis. The expression of p21, the cyclin dependent kinase inhibitor was increased in treated samples of both U87MG and U87-MCSF cells. Statistical significance is denoted by * (p<0.05), ** (p<0.01) and *** (p<0.001).

Cyclins involved in G1 phase and G1-S transition were examined by quantitative real time PCR analysis after treating synchronised cells (in G0/G1 phase) with 5-FU for 18 h. Similarly, the cyclins involved in late S phase and G2/M phase were quantified after 24 h of 5-FU treatment. Cyclin D1 expression remained unaltered between treated U87MG and treated U87-MCSF cells. After 18 h of 5-FU treatment, cyclin E expression was significantly decreased in treated U87-MCSF cells but not in treated U87MG cells (Figure 4). However, after 24 h of 5-FU treatment, down regulation in cyclin E expression was seen in treated samples of both U87MG and U87-MCSF cells (Figure S4 in File S1). This denoted that the response of U87-MCSF cells to 5-FU treatment was faster than U87MG cells. A slight decrease in expression of cyclin A2 was also observed in 5-FU treated U87-MCSF cells in comparison to 5-FU treated U87MG cells after 24 h of 5-FU treatment. The expressions of cyclin B1 and cyclin B2 were less in both treated samples, correlating with the less number of cells progressed to the G2/M phase. The expression of p21, a cyclin-dependent kinase inhibitor, was increased in the treated samples of both U87MG and U87-MCSF cells. Thus, differential expression of cyclins and the cdk inhibitor, p21 at various stages of cell cycle corroborated with the cell growth retardation in 5-FU treated U87-MCSF cells.

### Epithelial-mesenchymal transition (EMT) of U87-MCSF cells

**Figure 5:**
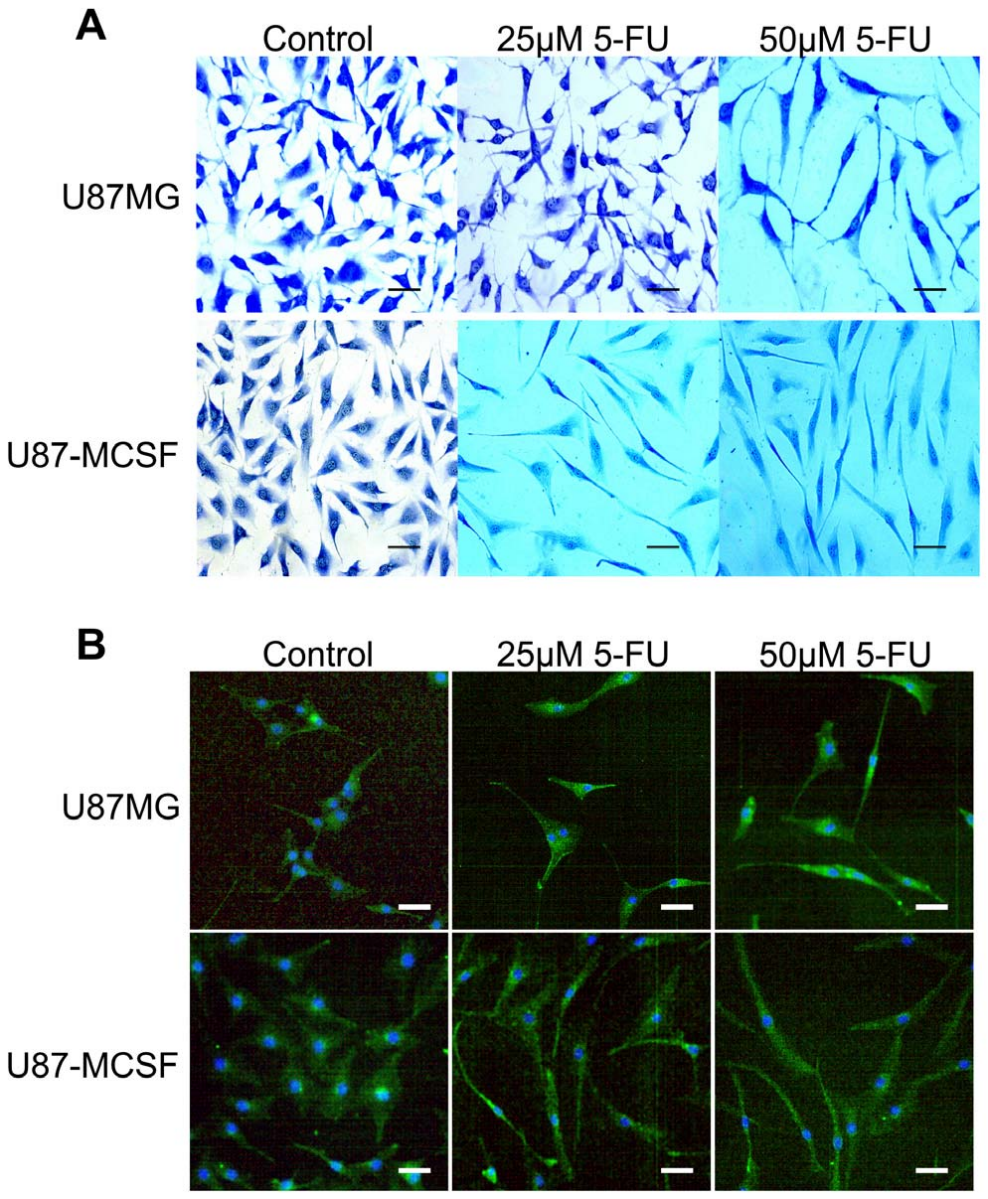
Microscopic examination of morphology of cells after 72-FU treatment. A. Methylene blue staining for detection of cell morphology after 5-FU treatment. B. Actin cytoskeleton staining of treated cells using anti β-actin antibody. The morphological pictures showed the presence of elongated and mesenchymal cells in U87-MCSF cells treated with 25 µM 5-FU but not in U87MG cells treated with 25 µM 5-FU. Upon 50 µM 5-FU treatment, elongated cells were seen in both treated U87MG and treated U87-MCSF cells. Scale bar: 50 µm.

**Figure 6:**
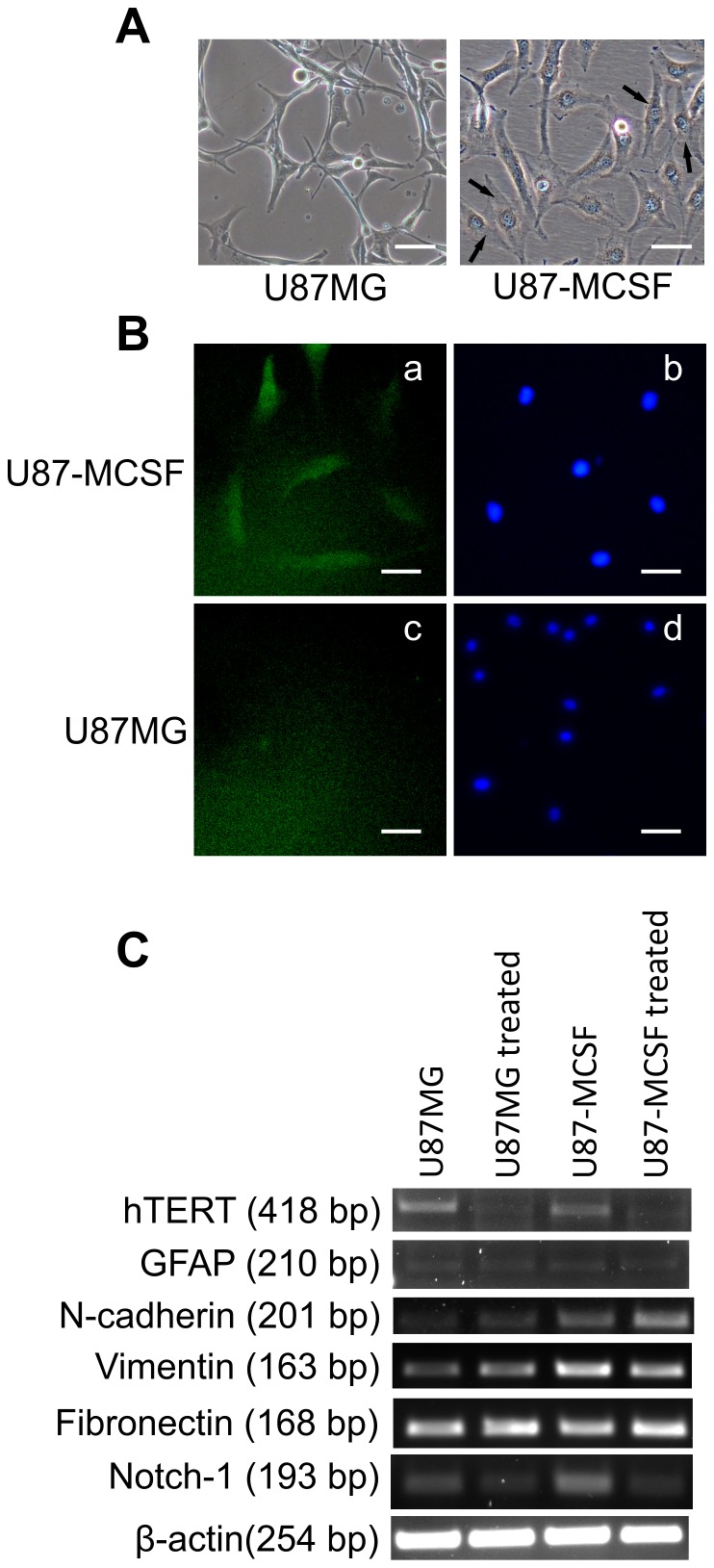
Analysis of MCSF localisation and EMT markers. A. Morphology of untreated U87MG and U87-MCSF cells. U87-MCSF cells showed spindle shaped, mesenchymal like cells (indicated by arrows). B. Microscopic studies using anti-MCSF antibody revealed the cytoplasmic location of MCSF. Triton X-100 was used as membrane perforating agent in fixing solution. Nucleus stained with DAPI were also shown. C. Semi quantitative RT-PCR analysis of hTERT, GFAP, N-cadherin, Vimentin, Fibronectin and Notch-1. Expression of mesenchymal cell markers, N-cadherin, vimentin and Notch-1 increased in U87-MCSF cells. Scale bar: 50 µm.

Apart from cell growth retardation, morphological changes were visible in methylene blue stained U87-MCSF cells treated with even low dose of 5-FU. Appearance of elongated, spindle shaped morphology with lengthy processes was seen in U87-MCSF cells with 25 µM 5-FU treatment for 72 h, but not in U87MG cells (Figure 5A). However, elongated morphology was seen in both cells with 50 µM 5-FU treatment. Further, cytoskeleton staining of untreated and treated samples of U87MG and U87-MCSF cells was done using anti β-actin antibody (Figure 5B). The results obtained reinforced the morphological changes observed in methylene blue staining. Elongated and mesenchymal cells were observed in 25 µM 5-FU treated U87-MCSF cells but not in 25 µM 5-FU treated U87MG cells after 72 h treatment. But, elongated cells were seen in both U87MG and U87-MCSF cells when treated with 50 µM 5-FU for 72 h. Additionally, microscopic examination of DAPI stained cells with 25 and 50 µM of 5-FU treatment after 120 h showed intact nuclei, and the calceinAM stained cells under same conditions, conferred elongated spindle shaped morphologies (Figure S5 in File S1), which was similar to the observation in methylene blue staining. Expression of the astrocytic differentiation marker, glial fibrillary acidic protein (GFAP) was absent and the expression of hTERT was significantly reduced in both U87MG and U87-MCSF cells after 5-FU treatment, justifying the suppression of cell proliferation (Figure 6C).

The presence of spindle shaped with more mesenchymal appearing cells in untreated U87-MCSF cells (Figure 6A) indicated the possible occurrence of epithelial-mesenchymal transition. The expression of epithelial cell marker E-cadherin and mesenchymal cell markers vimentin, N-cadherin, fibronectin was analysed by semi-quantitative RT-PCR. E-cadherin expression was not seen in untreated and treated cells of both U87MG and U87-MCSF cells (data not shown). However, there was a substantial increase in the expression of mesenchymal cell markers vimentin (∼2 fold in both untreated and treated samples) and N-cadherin (1.98 and 3.32 folds in untreated and treated samples, respectively) in U87-MCSF cells (Figure 6C). The expression level of fibronectin was unaltered in both treated cell types. Moreover the expression of Notch-1, the inducer of epithelial–mesenchymal transition (EMT), was found to be significantly upregulated (2.14 fold) in U87-MCSF cells. Microscopic examination of U87-MCSF cells, after fixing with 3.7% paraformaldehyde solution containing Triton X-100, revealed the cytoplasmic location of MCSF (Figure 6B). However, in the absence of Triton X-100 in the fixing solution no fluorescence was observed (data not shown) indicating the absence of MCSF at the cell membrane.

### Appearance of Cancer stem cell population and upregulation of ABC transporters

**Figure 7:**
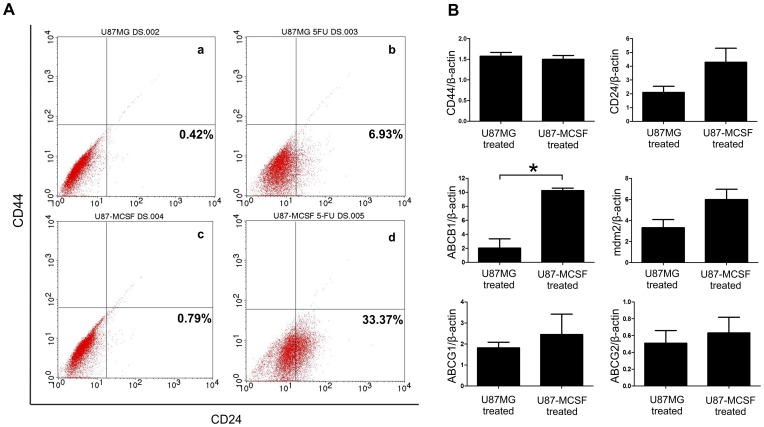
Analysis of markers of cancer stem cells and genes involved in drug resistance. A. Flow cytometry analysis for the expression of CD24 and CD44 in treated cells. B. Real time PCR analysis of expression of CD44, CD24, ABCB1, mdm2, ABCG1 and ABCG2. The data was plotted as the ratio of gene expression in treated sample to untreated sample for the respective cells. Statistical significance is denoted by * (p<0.05), ** (p<0.01) and *** (p<0.001).

The reduced proliferation, change in cell morphology and indications for the presence of EMT suggested the possible induction of cancer stem cells in U87-MCSF cells upon treatment with 5-FU. We analysed the expression of markers for cancer stem cells, CD24 and CD44 by flow cytometry and real time PCR. Flow cytometry analysis showed that the expression of CD24 was increased by 6.93% and 33.37% in U87MG cells and U87-MCSF cells respectively, when treated with 25 µM 5-FU (Figure 7A). No increase in CD44 expression was noted in treated samples of both U87MG and U87-MCSF cells. Quantitative expression analysis by real time PCR also revealed the increase in CD24 expression by around 4 fold in treated U87-MCSF cells, whereas only 2 fold increase in CD24 expression was seen in treated U87MG cells. CD44 expression remained unchanged in treated cells of both U87MG and U87-MCSF (Figure 7B).

The ABC transporter genes have been associated with the expulsion of drugs in many types of cancers. We analysed the expression of ABCG1, ABCG2 and ABCB1 by quantitative real time PCR. Figure 6B showed that the expression of ABCG1 and ABCB1 was increased in both treated U87MG and treated U87-MCSF cells. However, the expression of ABCB1 was increased by 10.2 fold in treated U87-MCSF cells as compared to the 2 fold increase in treated U87MG cells. Similarly, the expression of mdm2 oncogene was also increased by 6 fold in treated U87-MCSF cells in comparison to the 3.3 fold increase found in the treated U87MG cells. We also examined the expression level of RALBP1gene, a non-ABC transporter associated with MDR (multidrug resistance). We found a marginal increase in the expression of RALBP1 in untreated U87-MCSF cells (1.29 fold) as compared to the untreated U87MG cells (Figure S6 in File S1). However, upon 5-FU treatment, no upregulation in RALBP1 was seen in treated samples with respect to their corresponding untreated samples.

## Discussion

Glioblastoma remains one of the most prevalent malignant tumors with poor therapeutic response [11]. Expression of various multi-drug resistant proteins has mainly contributed to the ability of glioma cells to develop chemoresistant phenotype [12], [13]. In the present study, MCSF expressing U87MG glioblastoma cells exhibited reduced growth and significant retardation in cell proliferation without undergoing apoptosis after 5-FU administration. A balance in the expression of pro and anti-apoptotic genes determine the fate of the cells in entering apoptotic pathway [14]. Analysis of expression of pro and anti-apoptotic genes revealed that upregulation in the expression of pro-apoptotic gene, Bax is counter-balanced by the upregulation in the expression of anti-apoptotic Bcl-xL in both treated U87MG and treated U87-MCSF cells. However, treated U87-MCSF cells had lesser expression of pro-apoptotic gene Bax and higher expression of anti-apoptotic Bcl-xL as compared to treated U87MG cells. Our results showed that although treated samples of both U87MG and U87-MCSF cells did not undergo apoptosis, 5-FU treated U87-MCSF cells had more resistance to apoptosis than 5-FU treated U87MG cells.

We observed that MCSF is located in the cytoplasm and not at the cell membrane. Moreover, the expression of the receptor for MCSF, CSF1R was down regulated in U87-MCSF cells, which was in agreement with the previous report [15] about down regulation of mRNA of CSF1R by MCSF in primary macrophages. CSF1R was thought to function at the plasma membrane, but recent evidence showed the presence of functional receptor for MCSF at the nuclear envelope of various cancer cells and macrophages [16]. This raises the possibility that the observed effects of MCSF in U87-MCSF cells upon 5-FU treatment could be mediated through the receptor located at the nuclear envelope.

EMT is a conserved transdifferentiation cellular process conferring the features of mesenchymal cells over the basal epithelial cells, increasing their invasive properties and metastasis [17], [18]. MCSF expression in U87MG cells resulted in the appearance of spindle shaped, more mesenchymal type cells indicating the occurrence of EMT. A corresponding upregulation in expression of mesenchymal markers like N-cadherin and vimentin (1.98 and 2.18 fold respectively) was also noted in U87-MCSF cells. E-cadherin expression was found to be absent in all the samples analysed (data not shown) which correlated with the previously reported data showing lack of E-cadherin expression in glioblastoma [19]. Moreover, an increase in expression of notch-1 was observed in U87-MCSF cells. Upregulation of Notch-1 is implicated in inducing epithelial-mesenchymal transition (EMT), which has been previously reported to increase the invasive properties of pancreatic cancer cells [20].

With 5-FU treatment, the expression of the mesenchymal marker, N-cadherin was further increased in treated U87-MCSF cells by 3.32 fold while the expression of vimentin remained unchanged. In treated U87MG cells, only a marginal increase in the expression of N-cadherin and vimentin (1.26 and 1.41 fold respectively) was noted. 5-FU treatment induced mesenchymal properties in both U87MG and U87-MCSF cells as seen in Figure 5 and Figure 6. However, the concentration of 5-FU required to induce these changes varied between U87MG and U87-MCSF cells. While elongated and spindle shaped mesenchymal cells were seen in treated U87-MCSF when treated with 25 µM 5-FU for 72 h, mesenchymal morphology can be seen in treated U87MG cells only when treated with 50 µM 5-FU for 72 h. But when treatment with 25 µM 5-FU was prolonged for 120 h, elongated mesenchymal type cells were seen in treated samples of both U87MG and U87-MCSF cells (Figure S5 in File S1).

Recent reports established that EMT could trigger the acquisition of stem-like properties in the tumor cells [21]. A sub population of cells within several tumors exhibit cancer stem cell (CSC) properties with extremely slow rate of proliferation, increased expression of multidrug resistance genes and are considered to be the primary reason for the relapse of the tumor after treatment [22], [23]. Retardation in proliferation, occurrence of EMT and resistance to drug treatment suggested the presence of CSCs or their precursors in treated cell populations. Analysis of stemness-associated surface markers, CD24 and CD44 in the treated cells by flow cytometry depicted an increase in CD24^high^/CD44^low^ population of cells in both treated U87MG and treated U87-MCSF cells. However, treated U87-MCSF cells showed higher percentage (33.37%) of CD24^high^/CD44^low^ cells as compared to 6.93% in treated U87MG cells. In addition, quantitative expression analysis by real time PCR revealed that CD44 expression was uniform in 5-FU treated samples of both cells, but the CD24 expression was considerably higher in treated U87-MCSF cells. Previous reports showed that CSCs isolated from breast cancer were predominantly CD44^high^/CD24^low^ cells [21]. In contrast to this, breast cancer cells with CD44^low^/CD24^high^ phenotype was also reported to have poor prognosis with treatment [24]. Additionally, CD44^high^/CD24^high^ CSCs were found in gastric, pancreatic, ovarian and colorectal cancers [23], [25], [26]. Recently, Deng et al. [27] reported overexpression of CD24 in higher grade gliomas and the patients with CD24 positive tumors showed poor prognostic outcome after surgery. The definition of what exactly constitutes cancer stem cells remains unclear and there are no universally accepted cancer stem cell markers for different types of cancers [26]. Here, in our study we show the presence of CD24^high^/CD44^low^ cancer stem cells in treated samples of U87MG and U87-MCSF cells. However, the increased expression of CD24 surface marker exacerbated the cancer stem cell properties of treated U87-MCSF cells.

Tumor cells are known to acquire resistance to chemotherapeutic drugs through expression of various multidrug resistance genes and cancer stem cells can effectively increase the drug resistance through upregulation of drug efflux transporter genes [28]. CSCs isolated by forming spheroid cultures of glioblastoma tumor cells were previously shown to have high expression of ABCB1 gene and were found to be significantly resistant to chemotherapeutic agents [12]. Our results showed that U87MG cells acquired resistance to 5-FU through its conversion to cancer stem cells along with upregulation of mdm2 oncogene and ABC transporter genes, ABCB1 and ABCG1. The expression of MCSF in U87MG cells leading to cancer stem cell population further aggravated the resistive phenotype through an elevated increase in the expression of ABCB1 and mdm2 as compared to the treated U87MG cells. RALBP1 is a ubiquitous protein present in humans and is one of the non-ABC transporter proteins implicated in resistance to many chemotherapeutic drugs [29], [30]. In our study, although the expression of RALBP1 was slightly increased in untreated U87-MCSF cells as compared to untreated U87MG cells, no upregulation in RALBP1 expression was observed in 5-FU treated samples of both U87MG and U87-MCSF cells.

**Figure 8:**
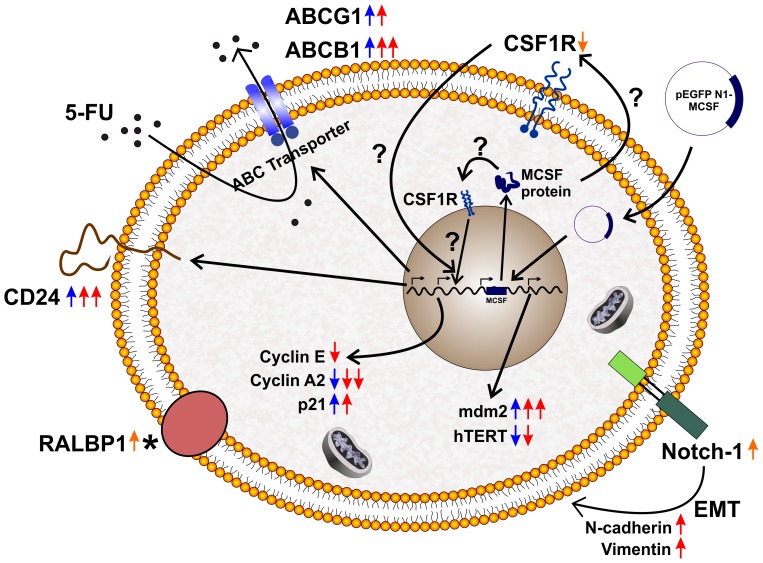
Schematic representation for the mechanism of 5-FU resistance in U87MG and U87-MCSF cells. The scheme depicts the upregulation and downregulation of various genes involved in increasing the resistance of U87MG and U87-MCSF cells to 5-FU. The emergence of cancer stem cells in the treated samples was also shown. (Orange colour arrows indicated untreated U87-MCSF cells; Blue colour arrows indicated 5-FU treated U87MG cells; Red colour arrows indicated 5-FU treated U87-MCSF cells; * indicated that RALBP1 expression remained unchanged in treated samples with respect to their corresponding untreated samples).

Taken together, our results demonstrated that U87MG cells developed resistive phenotype when subjected to 5-FU treatment. This drug resistance was further enhanced when MCSF gene was expressed in U87MG cells. MCSF was known to be a pro-tumoral cytokine. The elevated expression of MCSF along with its receptor CSF1R in breast, uterine and ovarian cancer was associated with tumor progression [31], [32]. Further, high level of MCSF in several tumors had been strongly associated with poor prognosis [33], [34].Our results provide evidence for MCSF increasing the resistance of tumor cells to 5-FU, by upregulating the expression of MDR associated genes. To the best of our knowledge, this is the first report implicating the cancer stem cells as a major nodal point for the MCSF associated drug resistance in a tumor cell type. The pivotal genes involved in resistance towards 5-FU treatment and the emergence of stem-like properties in treated U87-MCSF cells are depicted in Figure. 8

## Supporting Information

File S1 **Figures S1–S6 & Table S1.** Figure S1. Overexpression of MCSF did not induce 5-FU mediated apoptosis. (A–C) U87MG. (D–F) U87-MCSF. The results revealed that MCSF expression failed to induce apoptosis after treatment with 25 µM 5-FU (B, E) and 50 µM 5-FU (C, F) for five days. As U87MG cells were resistant to 5-FU and cisplatin, Hela cells were used as appropriate controls-(G) untreated HeLa cells and (H) HeLa cells treated with cisplatin. Figure S2. Semi-quantitative RT-PCR analysis of pro and anti-apoptotic genes in U87MG and U87-MCSF cells after 72 h treatment with 5-FU. Figure S3. Comparison of cell cycle between U87MG and U87-MCSF cells. No difference was seen in pattern of cell cycle between U87MG and U87-MCSF cells. Figure S4. RT-PCR analysis of expression of cyclin E after 24 h of 5-FU treatment. The results showed decrease in expression of cyclin E in treated samples of both U87MG and U87-MCSF cells. Figure S5. Microscopic examination by DAPI/CalceinAM dual staining after 120 h of 5-FU treatment. The results showed the presence of elongated cells in all the treated samples of U87MG and U87-MCSF cells. DAPI staining showed intact nuclei and absence of apoptosis. Scale bar: 50 µm. Figure S6. Semi-quantitative RT-PCR analysis of expression of RALBP1. A slight increase in expression of RALBP1 was observed in untreated U87-MCSF cells. However, no increase in RALBP1 expression was found after 5-FU treatment. Table S1. List of primers used.
(DOC)

## References

[1] Stanley ER, Berg KL, Einstein DB, Lee PS, Pixley FJ, et al (1997) Biology and action of colony–stimulating factor-1. Mol Reprod Dev 46: 4–10. 8981357 10.1002/(SICI)1098-2795(199701)46:1<4::AID-MRD2>3.0.CO;2-V

[2] Pollard JW, Hennighausen L (1994) Colony stimulating factor 1 is required for mammary gland development during pregnancy. Proc Natl Acad Sci U S A 91: 9312–9316. 7937762 10.1073/pnas.91.20.9312 PMC44802

[3] Michaelson MD, Bieri PL, Mehler MF, Xu H, Arezzo JC, et al (1996) CSF-1 deficiency in mice results in abnormal brain development. Development 122: 2661–2672. 8787741 10.1242/dev.122.9.2661

[4] Douglass TG, Driggers L, Zhang JG, Hoa N, Delgado C, et al (2008) Macrophage colony stimulating factor: not just for macrophages anymore! A gateway into complex biologies. Int Immunopharmacol 8: 1354–1376. 18687298 10.1016/j.intimp.2008.04.016

[5] Lewis CE, Pollard JW (2006) Distinct role of macrophages in different tumor microenvironments. Cancer Res 66: 605–612. 16423985 10.1158/0008-5472.CAN-05-4005

[6] Curry JM, Eubank TD, Roberts RD, Wang Y, Pore N, et al (2008) M-CSF signals through the MAPK/ERK pathway via Sp1 to induce VEGF production and induces angiogenesis in vivo. PLoS One 3: e3405. 18852899 10.1371/journal.pone.0003405 PMC2566603

[7] Jadus MR, Chen Y, Boldaji MT, Delgado C, Sanchez R, et al (2003) Human U251MG glioma cells expressing the membrane form of macrophage colony-stimulating factor (mM-CSF) are killed by human monocytes in vitro and are rejected within immunodeficient mice via paraptosis that is associated with increased expression of three different heat shock proteins. Cancer Gene Ther 10: 411–420. 12719711 10.1038/sj.cgt.7700583

[8] Hoa NT, Zhang JG, Delgado CL, Myers MP, Callahan LL, et al (2007) Human monocytes kill M-CSF-expressing glioma cells by BK channel activation. Lab Invest 87: 115–129. 17318194 10.1038/labinvest.3700506

[9] Kawakami Y, Nagai N, Ohama K, Zeki K, Yoshida Y, et al (2000) Macrophage-colony stimulating factor inhibits the growth of human ovarian cancer cells in vitro. Eur J Cancer 36: 1991–1997. 11000582 10.1016/s0959-8049(00)00282-3

[10] Das AB, Loying P, Bose B (2012) Human recombinant Cripto-1 increases doubling time and reduces proliferation of HeLa cells independent of pro-proliferation pathways. Cancer Lett 318: 189–198. 22182448 10.1016/j.canlet.2011.12.013

[11] Sharma S, Chockalingam S, Sanpui P, Chattopadhyay A, Ghosh SS (2013) Silver Nanoparticles Impregnated Alginate-Chitosan-Blended Nanocarrier Induces Apoptosis in Human Glioblastoma Cells. Adv Healthcare Mater. doi:10.1002/adhm.201300090. 10.1002/adhm.201300090 23852919

[12] Nakai E, Park K, Yawata T, Chihara T, Kumazawa A, et al (2009) Enhanced MDR1 expression and chemoresistance of cancer stem cells derived from glioblastoma. Cancer Invest 27: 901–908. 19832037 10.3109/07357900801946679

[13] Bredel M, Zentner J (2002) Brain-tumour drug resistance: the bare essentials. The Lancet Oncology 3: 397–406. 12142169 10.1016/s1470-2045(02)00786-6

[14] Finkel E (2001) The mitochondrion: is it central to apoptosis? Science 292: 624–626. 11330312 10.1126/science.292.5517.624

[15] Yue X, Favot P, Dunn TL, Cassady AI, Hume DA (1993) Expression of mRNA encoding the macrophage colony-stimulating factor receptor (c-fms) is controlled by a constitutive promoter and tissue-specific transcription elongation. Mol Cell Biol 13: 3191–3201. 8497248 10.1128/mcb.13.6.3191-3201.1993 PMC359760

[16] Zwaenepoel O, Tzenaki N, Vergetaki A, Makrigiannakis A, Vanhaesebroeck B, et al (2012) Functional CSF-1 receptors are located at the nuclear envelope and activated via the p110delta isoform of PI 3-kinase. FASEB J 26: 691–706. 22084313 10.1096/fj.11-189753

[17] Sethi N, Kang Y (2011) Notch signalling in cancer progression and bone metastasis. Br J Cancer 105: 1805–1810. 22075946 10.1038/bjc.2011.497 PMC3251892

[18] Singh A, Settleman J (2010) EMT, cancer stem cells and drug resistance: an emerging axis of evil in the war on cancer. Oncogene 29: 4741–4751. 20531305 10.1038/onc.2010.215 PMC3176718

[19] Bellail AC, Hunter SB, Brat DJ, Tan C, Van Meir EG (2004) Microregional extracellular matrix heterogeneity in brain modulates glioma cell invasion. Int J Biochem Cell Biol 36: 1046–1069. 15094120 10.1016/j.biocel.2004.01.013

[20] Bao B, Wang Z, Ali S, Kong D, Li Y, et al (2011) Notch-1 induces epithelial-mesenchymal transition consistent with cancer stem cell phenotype in pancreatic cancer cells. Cancer Lett 307: 26–36. 21463919 10.1016/j.canlet.2011.03.012 PMC3104092

[21] Mani SA, Guo W, Liao MJ, Eaton EN, Ayyanan A, et al (2008) The epithelial-mesenchymal transition generates cells with properties of stem cells. Cell 133: 704–715. 18485877 10.1016/j.cell.2008.03.027 PMC2728032

[22] Roesch A, Fukunaga-Kalabis M, Schmidt EC, Zabierowski SE, Brafford PA, et al (2010) A temporarily distinct subpopulation of slow-cycling melanoma cells is required for continuous tumor growth. Cell 141: 583–594. 20478252 10.1016/j.cell.2010.04.020 PMC2882693

[23] Dembinski JL, Krauss S (2009) Characterization and functional analysis of a slow cycling stem cell-like subpopulation in pancreas adenocarcinoma. Clin Exp Metastasis 26: 611–623. 19421880 10.1007/s10585-009-9260-0 PMC2776152

[24] Ahmed MA, Aleskandarany MA, Rakha EA, Moustafa RZ, Benhasouna A, et al (2012) A CD44(-)/CD24(+) phenotype is a poor prognostic marker in early invasive breast cancer. Breast Cancer Res Treat 133: 979–995. 22119938 10.1007/s10549-011-1865-8

[25] Zhang C, Li C, He F, Cai Y, Yang H (2011) Identification of CD44+CD24+ gastric cancer stem cells. J Cancer Res Clin Oncol 137: 1679–1686. 21882047 10.1007/s00432-011-1038-5 PMC11828146

[26] Jaggupilli A, Elkord E (2012) Significance of CD44 and CD24 as cancer stem cell markers: an enduring ambiguity. Clin Dev Immunol 2012: 708036. 22693526 10.1155/2012/708036 PMC3369436

[27] Deng J, Gao G, Wang L, Wang T, Yu J, et al. (2012) CD24 Expression as a Marker for Predicting Clinical Outcome in Human Gliomas. Journal of Biomedicine and Biotechnology 2012. 10.1155/2012/517172 PMC3303885 22500096

[28] Dean M, Fojo T, Bates S (2005) Tumour stem cells and drug resistance. Nat Rev Cancer 5: 275–284. 15803154 10.1038/nrc1590

[29] Drake KJ, Singhal J, Yadav S, Nadkar A, Pungaliya C, et al (2007) RALBP1/RLIP76 mediates multidrug resistance. Int J Oncol 30: 139–144. 17143522

[30] Awasthi S, Hallene KL, Fazio V, Singhal SS, Cucullo L, et al (2005) RLIP76, a non-ABC transporter, and drug resistance in epilepsy. BMC Neurosci 6: 61. 16188027 10.1186/1471-2202-6-61 PMC1249579

[31] Kacinski BM (1997) CSF-1 and its receptor in breast carcinomas and neoplasms of the female reproductive tract. Mol Reprod Dev 46: 71–74. 8981366 10.1002/(SICI)1098-2795(199701)46:1<71::AID-MRD11>3.0.CO;2-6

[32] Smith HO, Anderson PS, Kuo DY, Goldberg GL, DeVictoria CL, et al (1995) The role of colony-stimulating factor 1 and its receptor in the etiopathogenesis of endometrial adenocarcinoma. Clin Cancer Res 1: 313–325. 9815987

[33] Chambers SK, Kacinski BM, Ivins CM, Carcangiu ML (1997) Overexpression of epithelial macrophage colony-stimulating factor (CSF-1) and CSF-1 receptor: a poor prognostic factor in epithelial ovarian cancer, contrasted with a protective effect of stromal CSF-1. Clin Cancer Res 3: 999–1007. 9815777

[34] Mroczko B, Groblewska M, Wereszczynska-Siemiatkowska U, Okulczyk B, Kedra B, et al (2007) Serum macrophage-colony stimulating factor levels in colorectal cancer patients correlate with lymph node metastasis and poor prognosis. Clin Chim Acta 380: 208–212. 17368603 10.1016/j.cca.2007.02.037

