# Supplementary material for: Amelioration of Cancer Stem Cells in Macrophage Colony Stimulating Factor-Expressing U87MG-Human Glioblastoma upon 5-Fluorouracil Therapy
Source: PLoS One. 2013 Dec 31;8(12):e83877. doi: 10.1371/journal.pone.0083877 (PMC3877109; doi:10.1371/journal.pone.0083877)
Supplement: File S1 — Figures S1–S6 & Table S1. Figure S1. Overexpression of MCSF did not induce 5-FU mediated apoptosis. (A–C) U87MG. (D–F) U87-MCSF. The results revealed that MCSF expression failed to induce apoptosis after treatment with 25 µM 5-FU (B, E) and 50 µM 5-FU (C, F) for five days. As U87MG cells were resistant to 5-FU and cisplatin, Hela cells were used as appropriate controls-(G) untreated HeLa cells and (H) HeLa cells treated with cisplatin. Figure S2. Semi-quantitative RT-PCR analysis of pro and anti-apoptotic genes in U87MG and U87-MCSF cells after 72 h treatment with 5-FU. Figure S3. Comparison of cell cycle between U87MG and U87-MCSF cells. No difference was seen in pattern of cell cycle between U87MG and U87-MCSF cells. Figure S4. RT-PCR analysis of expression of cyclin E after 24 h of 5-FU treatment. The results showed decrease in expression of cyclin E in treated samples of both U87MG and U87-MCSF cells. Figure S5. Microscopic examination by DAPI/CalceinAM dual staining after 120 h of 5-FU treatment. The results showed the presence of elongated cells in all the treated samples of U87MG and U87-MCSF cells. DAPI staining showed intact nuclei and absence of apoptosis. Scale bar: 50 µm. Figure S6. Semi-quantitative RT-PCR analysis of expression of RALBP1. A slight increase in expression of RALBP1 was observed in untreated U87-MCSF cells. However, no increase in RALBP1 expression was found after 5-FU treatment. Table S1. List of primers used. (DOC) [file pone.0083877.s001.doc]

**Supplementary Information**

**Amelioration of Cancer Stem Cells in Macrophage Colony Stimulating Factor-Expressing U87MG-Human Glioblastoma upon 5-Fluorouracil Therapy**

S. Chockalingam^1^ and Siddhartha Sankar Ghosh^1,2^

^1^Department of Biotechnology,

Indian Institute of Technology Guwahati, Guwahati, Assam, India.

^2^Centre for Nanotechnology,

Indian Institute of Technology Guwahati, Guwahati, Assam, India.

To whom correspondence should be addressed: Siddhartha Sankar Ghosh, Department of Biotechnology and Centre for Nanotechnology, Indian Institute of Technology Guwahati, Guwahati, Assam, India. Tel: 91-361-258-2206; Email: [sghosh@iitg.ernet.in](mailto:sghosh@iitg.ernet.in).

**Supplementary methods**

**Determination of Mitochondrial Membrane Potential (MMP)**

Briefly, 5x10^4^ cells per well of six well plate were treated with 5-FU and then trypsinized, centrifuged and resuspended in complete medium containing 10 µg/ml JC-1. The cell suspension was kept in dark for 10 min at room temperature, washed twice with PBS, resuspended in 500 µl PBS and analysed with a flow cytometer (FacsCalibur, BD Biosciences, NJ) with the CellQuest Pro software.

**DAPI/CalceinAM staining**

After drug treatment for 120 h, cells were washed thoroughly with PBS and stained with 2.5 µM calceinAM solution for 15 min in dark. Then, cells were washed twice and incubated with media containing 300nM DAPI for 3 min, finally washed and viewed under fluorescence microscope (Nikon ECLIPSE T*i*-U, Japan) with an excitation filter of 480/15 nm(for calceinAM) and 360/20 nm (for DAPI).


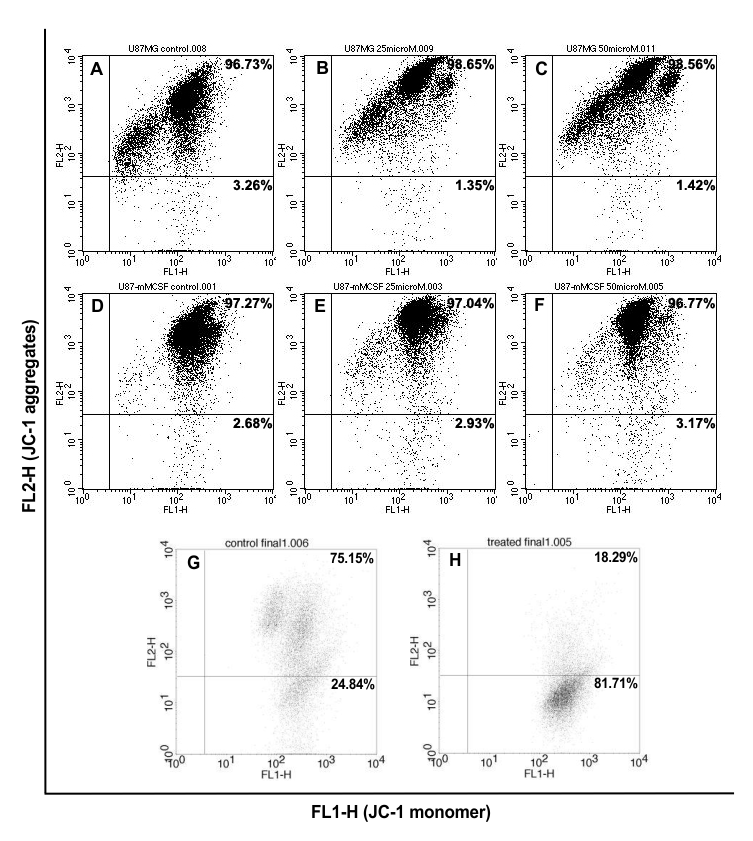


**Figure S1**


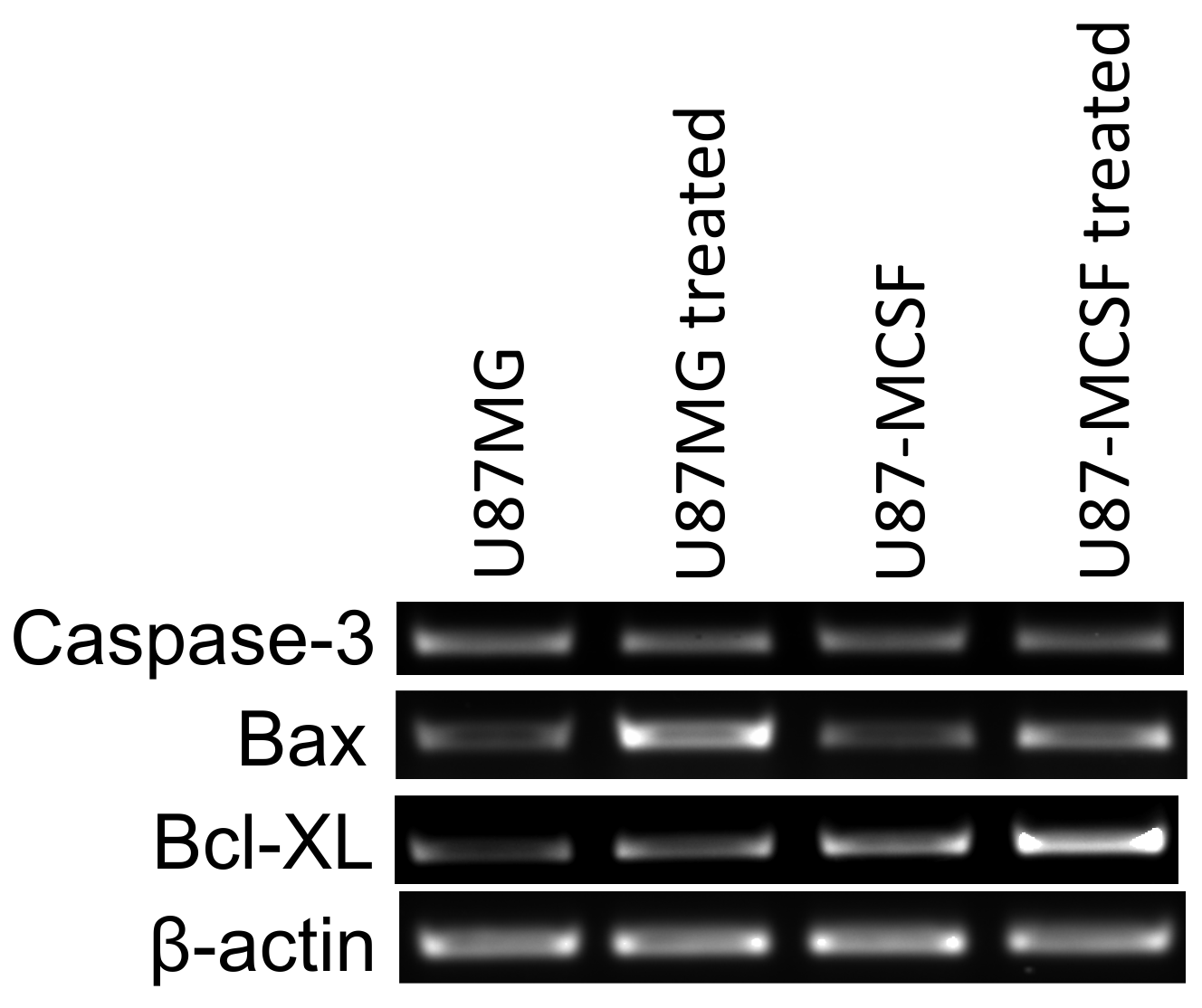


**Figure S2**


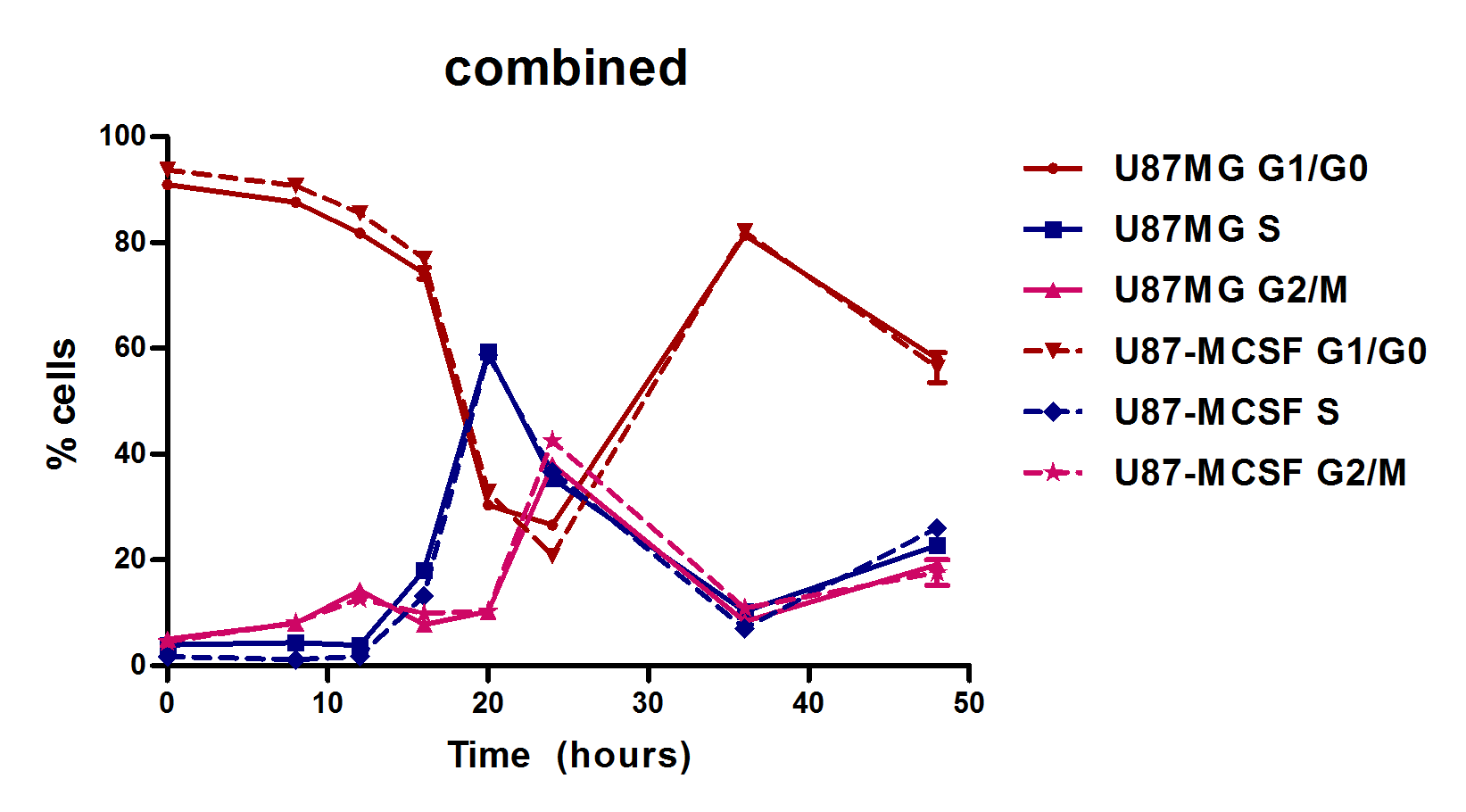


**Figure S3**


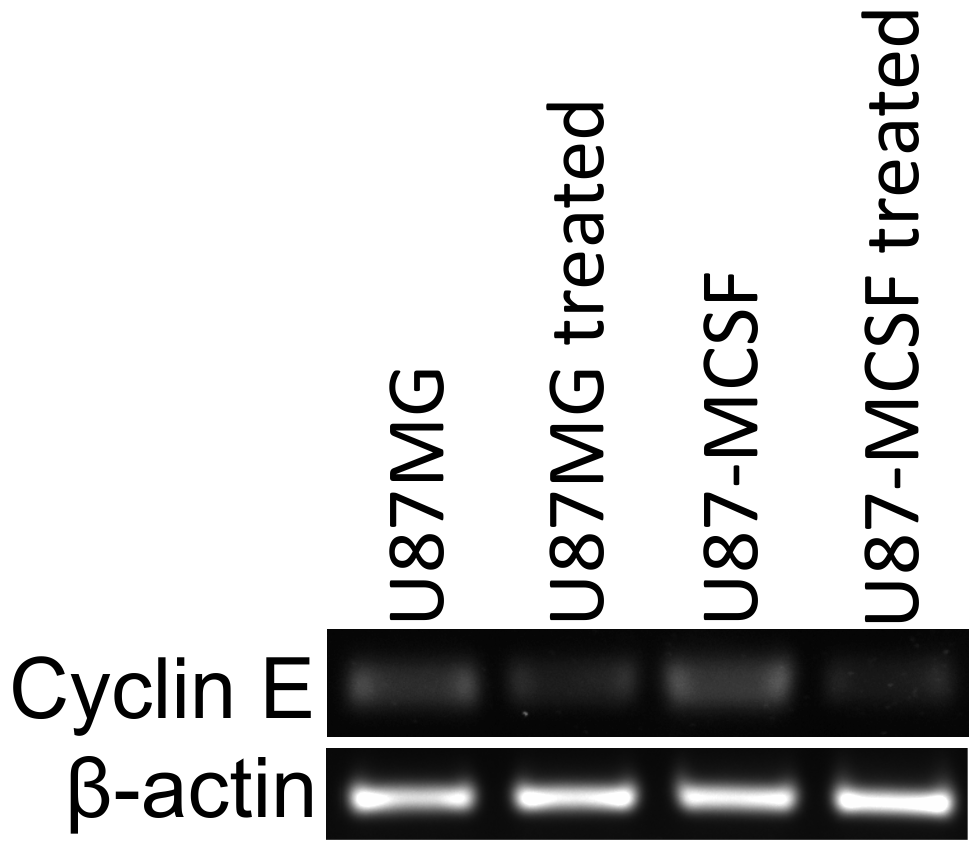


**Figure S4**

**
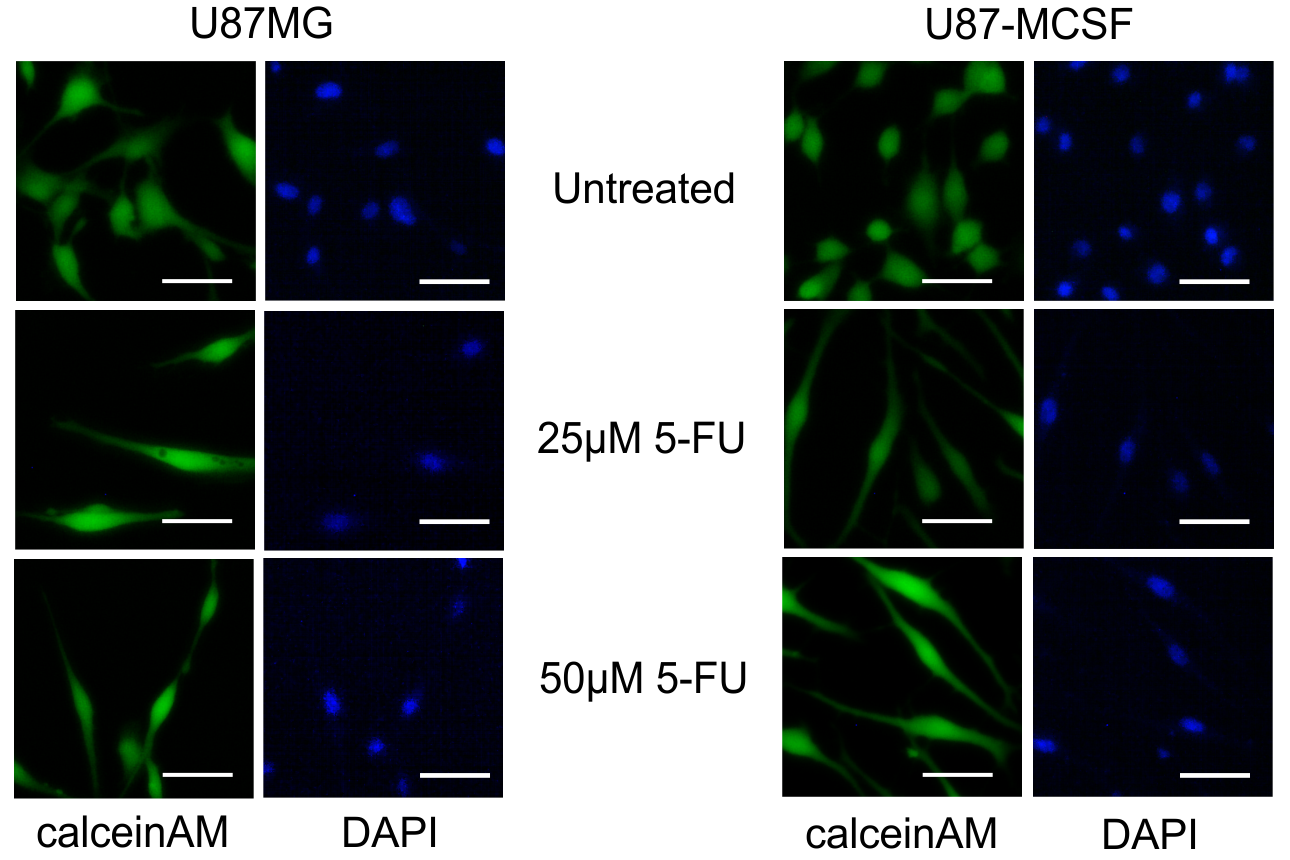
**

**Figure S5**


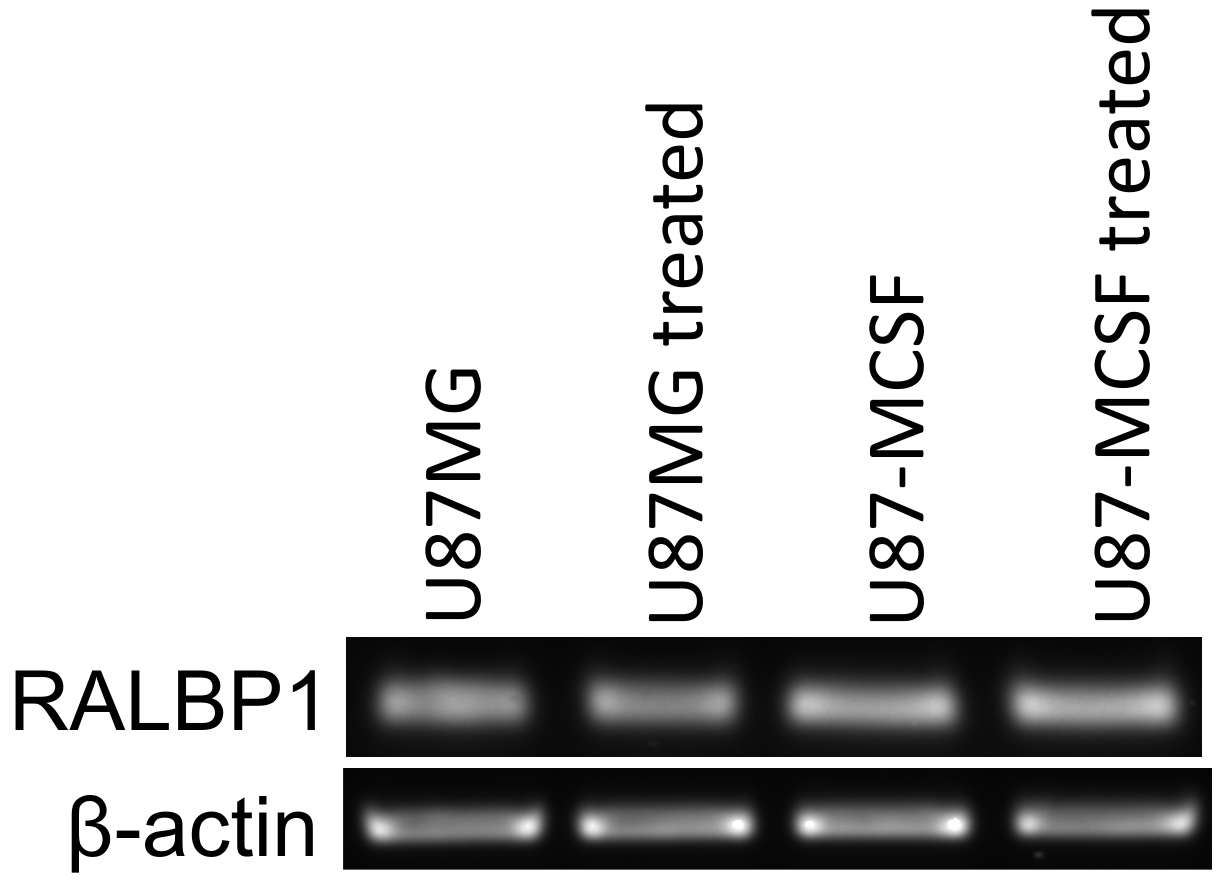


**Figure S6**

**Table S**1

| **Gene** | **Primer sequence** |
| --- | --- |
| β-actin | Forward: 5`-CTGTCTGGCGGCACCACCAT-3`  Reverse: 5`-GCAACTAAGTCATAGTCCGC-3` |
| MCSF | Forward: 5`-GGAATTCCCATGACCGCGCCGGGCGCC-3`  Reverse: 5`-AAGGGCCCCACTGGCAGTTCCAC-3` |
| CSF1R | Forward: 5`- CTGGAGCTCCGGGACCTGCT-3`  Reverse: 5`- CCACTTCACAGGCAGGCGGG-3` |
| GFAP | Forward: 5`- CGGCTCGATCAACTCA-3`  Reverse: 5′-CTCCTCCAGCGACTCAAT-3′ |
| hTERT | Forward: 5′-GTACATGCGACAGTTC-3′  Reverse: 5′-TTCTACAGGGAAGTTCAC-3′ |
| CyclinD1 | Forward: 5`-CGCCCCACCCCTCCAG-3`  Reverse: 5`-CGCCCAGACCCTCAGACT-3` |
| CyclinE | Forward: 5`-CCACACCTGACAAAGAAGATGATGAC-3`  Reverse: 5`-GAGCCTCTGGATGGTGCAATAAT-3` |
| CyclinA2 | Forward: 5`-ACGGCGCTCCAAGAGGACCA-3`  Reverse: 5`-AGCCAGGGCATCTTCACGCT-3` |
| CyclinB1 | Forward: 5`-TCTGGATAATGGTGAATGGACA-3`  Reverse: 5`-CGATGTGGCATACTTGTTCTTG-3` |
| CyclinB2 | Forward: 5`-AAAGTTGGCTCCAAAGGGTCCTT-3`  Reverse: 5`-GAAACTGGCTGAACCTGTAAAAAT-3 |
| mdm2 | Forward: 5′-CAGCAGGAATCATCGGACTCA-3′  Reverse: 5′-CCTTATTACACACAGAGCCAGGC-3′ |
| ABCB1 | Forward: 5`-CCCATCATTGCAATAGCAGG-3`  Reverse: 5` -TGTTCAAACTTCTGCTCCTGA-3` |
| ABCG1 | Forward: 5′- CAGGAAGATTAGACACTGTGG -3′  Reverse: 5′- GAAAGGGGAATGGAGAGAAGA-3′ |
| ABCG2 | Forward: 5′- CCGCGACAGTTTCCAATGACCT -3′  Reverse: 5′- GCCGAAGAGCTGCTGAGAACTGTA -3′ |
| CD44 | Forward: 5’-AGAAGGTGTGGGCAGAAGAA-3’  Reverse: 5’-AAATGCACCATTTCCTGAGA-3’ |
| CD24 | Forward: 5` CCCACGCAGATTTATTCCAG 3`  Reverse: 5` GACTTCCAGACGCCATTTG 3` |
| Notch-1 | Forward: 5′- TCCACCAGTTTGAATGGTCA -3′  Reverse: 5′- AGCTCATCATCTGGGACAGG -3′ |
| E-cadherin | Forward: 5` TGCCCAGAAAATGAAAAAGG 3`  Reverse: 5` GTGTATGTGGCAATGCGTTC 3` |
| N-cadherin | Forward: 5` ACAGTGGCCACCTACAAAGG 3`  Reverse: 5` CCGAGATGGGGTTGATAATG 3` |
| Fibronectin | Forward: 5` CAGTGGGAGACCTCGAGAAG 3`  Reverse: 5` TCCCTCGGAACATCAGAAAC 3` |
| Vimentin | Forward: 5` GAGAACTTTGCCGTTGAAGC 3`  Reverse: 5` GCTTCCTGTAGGTGGCAATC 3` |
| Caspase-3 | Forward: 5`-TTTGTTTGTGTGCTTCTGAGCC-3`  Reverse: 5`-ATTCTGTTGCCACCTTTCGG-3` |
| Bax | Forward: 5`-AAGCTGAGCGAGTGTCTCAAGCGC-3`  Reverse: 5`-TCCCGCCACAAAGATGGTCACG-3` |
| Bcl-xL | Forward: 5`-ATGGCAGCAGTAAAGCAAGCGC-3`  Reverse: 5`-TTCTCCTGGTGGCAATGGCG-3` |
| RalBP1 | Forward: 5`-ACTGTGCAGATCAGCAATCG-3`  Reverse: 5`-CCTGATCTCCTCCTTGATGC-3` |
